# Why morphology matters: the negative consequences of hasty descriptions of putative novelties in asexual ascomycetes

**DOI:** 10.1186/s43008-021-00073-z

**Published:** 2021-09-23

**Authors:** Ondřej Koukol, Gregorio Delgado

**Affiliations:** 1 Department of Botany, Faculty of Science, Charles University, Benátská 2, 128 01 Prague, Czech Republic; 2 EMLab P&K Houston, 10900 Brittmoore Park Dr. Suite G, Houston, TX 77041 USA

**Keywords:** Five new taxa, Asexual *Ascomycota*, *Dothideomycetes*, *Sordariomycetes*

## Abstract

Recent progress in the discovery of fungal diversity has been enabled by intensive mycological surveys in centres of global biodiversity. Descriptions of new fungal species have been almost routinely based on phenotypic studies coupled with single or multigene phylogenetic analyses of DNA sequence data. However, high accessibility of sequencing services together with an increasing amount of available molecular data are providing easier and less critical support for taxonomic novelties without carefully studying the phenotype, particularly morphology. As a result, the accelerated rate of species descriptions has been unfortunately accompanied by numerous cases of overlooking previously described and well documented species, some of them that have been known for more than a century. Here, we critically examined recent literature, phenotypic and molecular data, and detected multiple issues with putative novelties of asexual *Ascomycota* traditionally known as hyphomycetes. In order to fix these taxonomic problems, three new combinations within the genera *Pleopunctum*, *Camposporium* and *Sporidesmium*, and two new names in *Camposporium* are proposed. Moreover, three genera, *Aquidictyomyces*, *Fusiconidium* and *Pseudohelminthosporium*, together with nine species are reduced to synonymy. The examples outlined here clearly show the relevance of morphology in modern phylogenetic studies and the importance of more stringent ‘quality controls’ during biodiversity studies documenting the extensive fungal diversity in a speedy manner.

## INTRODUCTION

In recent years, the attempts to describe the “amazing fungal diversity” and to “flatten the curve” of known fungal species (Hyde et al. 2018, Hyde et al., 2020b) have led to an increased rate in the description of novel fungal taxa, especially in the Paleotropics but particularly in Southeast Asia. According to the 2020 *State of the World’s Plants and Fungi* report (Antonelli et al. 2020), 41% of species newly described in 2019 originated from Asia due to both an enormous fungal diversity hidden in this part of the world but also thanks to the diligent contribution and sustained efforts of local taxonomists. However, not all recent descriptions are well supported by phenotypic and molecular data and many of them are even questionable if not erroneous (Koukol et al. 2020). Here, we present multiple examples of recent issues with putative taxonomic novelties of asexual fungi belonging to *Ascomycota* traditionally known as hyphomycetes whenever literature records and phenotypic data provide sufficient evidence. Our intention is to call the mycological community involved in conducting fungal diversity studies for a reflection on the necessity of more critical and careful approaches during the introduction and description of novel microfungi. This call is made in the same positive spirit of Gams (2016) or Van Vooren and Vega (2018), for example, and in the best interest of all users of fungal names and the science of Mycology.

Species concepts in *Fungi* reflect their multiple evolutionary histories, huge diversity of morphology, trophic and reproductive strategies and have both practical and theoretical limitations (Lücking et al. 2020). Hyde et al. (2020b) recently emphasized that in current mycological praxis “we mostly rely on phenotypic and phylogenetic aspects and consider a species as a group of individuals sharing similar phenotypes and sufficient DNA similarities”. They further added that these individuals “must be sufficiently distinct from another sister group / species and have ample differences in DNA, supported with phylogeny or other analyses”. These simple although critical statements contain crucial assertions that are currently not followed in many studies, e.g., “group of individuals”. Although a single individual is extremely difficult if not impossible to define among microfungi, this can be avoided by studying multiple specimens or strains from different collection sites or substrates. However, a large number of asexual taxa in *Ascomycota* have been recently described based only on a single collection or strain, preventing a proper assessment of their species boundaries or intraspecific variability and the confirmation of taxonomic conclusions. Clearly, independent collections may be difficult to obtain for rare species, but descriptions may be postponed during long-term studies of a particular area or substrate until more specimens or strains become available.

Besides having limited representative material, too much emphasis is often placed on molecular data whereas a detailed study of the morphology together with a thorough review of relevant literature are often neglected. This unfortunate trend may be best viewed from the Notes sections following description of novelties, where sequence similarities are considered first if not the sole distinguishing characters used (Tibpromma et al. 2018). Although recent progress in successfully sequencing fungarium specimens has provided better delimitation of species boundaries even for “old” taxa (Forin et al. 2018), asexual ascomycetes in particular are often lacking reference ex-type sequences for comparison. Additionally, a huge gap in sequence availability for members of several thousand genera currently considered *Ascomycota “*incertae sedis” still exists (Wijayawardene et al. 2018). Therefore, the knowledge of morphology remains essential for proper delimitation of known species and description of taxonomic novelties (Aime et al. 2021).

## TAXONOMY

### Descriptions of recent *Hermatomyces* species suffer from inconsistencies

An illustrative example of multiple taxonomic issues is the genus *Hermatomyces*, which has received much attention in recent years from both the Paleotropics and Neotropics. Recently, an alarming high number of imprecise species descriptions based on mixed phenotypic and molecular data together with insufficient understanding of previous species delimitations was revealed (Koukol et al. 2018; Koukol and Delgado 2019; Delgado et al. 2020). Six out of the 28 binomial names introduced in *Hermatomyces* (Index Fungorum http://www.indexfungorum.org/) were found to be later synonyms and other three are considered doubtful or described based on insufficient evidence, which gives an error rate of 32% of erroneously described species in a single genus. One of the reasons of this high rate is that many recently described species such as *H. clematidis*, *H. bauhiniae,* and *H. trangensis* amongst others, were introduced based on a single or at most two collections from the same site and host. *Hermatomyces clematidis,* for example, described from a dead stem of *Clematis sikkimensis* in Thailand (Phukhamsakda et al. 2020) is morphologically identical with *Hermatomyces* sp. IMI 289491 collected from a dead twig in Ethiopia and recently studied by Koukol and Delgado (2019). A more complete overview on the ecology and the intraspecific variability of this species, for example the intensity of melanization in the apex of cylindrical conidia, would be obtained by a comparison with the African material and its distribution in this continent even in the absence of DNA sequence data. Additionally, hasty introduction of species descriptions has led to mishandling with own data. Delgado et al. (2020), for example, already showed that *H. bauhiniae* was based on a mixed collection and the published morphological description referred to a contaminating fungus. Ironically, the contaminant was described later from Thailand by the same authors in the same year as *Pleopunctum clematidis*. As Delgado et al. (2020) already suggested, a new combination is necessary to fix this name and therefore the confusing name *H. bauhiniae* is considered superfluous and a new combination is provided herein.

***Pleopunctum bauhiniae*** (Phukhams. et al.) Koukol & G. Delgado, **comb. nov.**

MycoBank: MB838824.

*Basionym*: *Hermatomyces bauhiniae* Phukhams. et al., *Fungal Diversity* **96**: 40 (2019).

*Synonym: Pleopunctum clematidis* Phukhams et al., *Fungal Diversity* **102**: 73 (2020).

*Description:* Hyde et al. (2019).

### *Canalisporium dehongense* and *C. thailandense* are synonyms

**Fig. 1:**
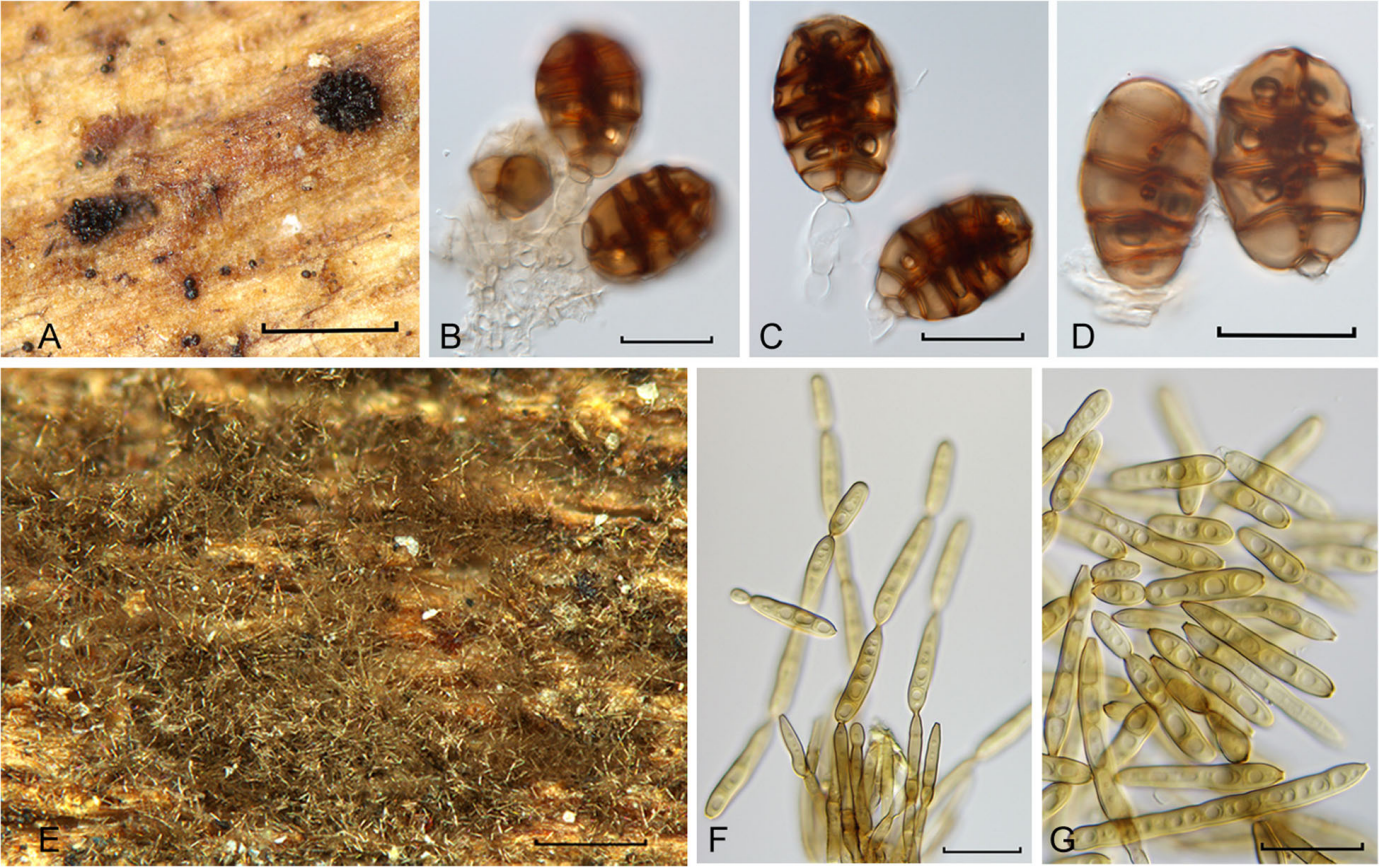
*Canalisporium caribense* (PRM 831526, holotype). **A.** Two pulvinate sporodochia on natural substrate. **B**, **C.** Conidia still attached to a conidiogenous cell. **D.** Detached conidia showing septal pores. *Sporidesmium tetracoilum* (PRC 4681). **E.** Colony on natural substrate. **F.** Chains of conidia produced on conidiogenous cells. **G.** Conidia. Scale bars **A**, **E** = 250 μm, **B**–**D**, **F**, **G** = 20 μm

The hasty description of novel species without critical evaluation of literature has also caused incongruences in the genus *Canalisporium*. Two species, *C. thailandense* and *C. krabiense*, were recently described based on single isolates obtained from leaves of *Pandanus* sp. in Thailand (Tibpromma et al., 2018). Although they differ substantially in morphology, sequences of ITS and LSU rDNA originating from their holotypes and deposited in GenBank are 100 and 99.8% identical, respectively. Surprisingly, their close position in a single clade in the phylogenetic tree was not commented by Tibpromma et al. (2018). Furthermore, only pairwise identities with sequences already available in GenBank were mentioned but not between the two species. Cross-contamination with DNA of *C. krabiense* obviouly resulted in identical sequences being connected with different “morphospecies”. Once the morphology of *C. thailandense* with its unique “globose to oval conidiogenous cells connected in a chain” is taken into consideration, another species described from China a year later (Hyde et al. 2019) could be considered conspecific. *Canalisporium dehongense* is characterized by the same chains of vesiculate cells with nearly identical conidial dimensions, but it has a distinct basal position within the *Canalisporium* lineage (Hyde et al., 2019). Interestingly, chains of hyaline globose to subglobose cells were repeatedly observed in specimens identified as *C. caribense*. To disentangle this conflict in morphological delimitation of *C. caribense*, we examined the holotype and confirmed the absence of these structures (Fig. 1B–C). Therefore, collections of this species in Goh et al. (1998) and Dayarathne et al. (2019) (collection MFLU15–3581, erroneously mentioned as the holotype) represent potential further records of *C. thailandense* worldwide. As *C. dehongense* is a later synonym of *C. thailandense*, the following synonymy is proposed:

***Canalisporium thailandense*** Tibpromma & K.D. Hyde, *Fungal Diversity* **93**: 122 (2018).

*Synonym: Canalisporium dehongense* W. Dong et al., *Fungal Diversity* **96**: 159 (2019).

In the most recent comprehensive treatment of the genus *Canalisporium*, Goh and Kuo (2021) accepted the recently introduced species *C. krabiense*, *C. thailandense* and *C. dehongense*. They also noted the obvious morphological similarity between *C. thailandense* and *C. dehongense* despite their distant phylogenetic position. However, given that both species are currently represented by a single specimen, our explanation for their similarity outlined above is more reasonable.

*Specimen examined*: **Cuba**, Isla de la Juventud (=Isla de Pinos), Cerro de San Juan, south-east of Santa Fe (now La Fe); on dead rotten branch, 22. Jan. 1981, *V. Holubová-Jechová* (PRM 831526, holotype of *Berkleasmium caribense* Hol.-Jech.).

### *Pseudohelminthosporium* is a synonym of *Neopodoconis*

The overemphasis on molecular data compounded with ignorance of old or recent mycological literature unnecessarily leads to further need for synonymies at the species level but also at the generic level. The recently introduced generic name *Pseudohelminthosporium* (Phukhamsakda et al. 2020) is a good example. Its type species, *Ps. clematidis*, described from a single specimen collected on dead stems of *Clematis sikkimensis* in Thailand, is obviously conspecific with the distinct and well documented species *Exosporium ampullaceum* (Ellis 1961). Its known distribution is pantropical and it has been recorded from several other Paleotropical countries so its presence in Thailand is not surprising. They both share a polytretic, sympodial conidiogenesis with cicatrized pores bearing dark and prominent scars. Conidia are obclavate, often rostrate, euseptate, sometimes smooth but usually verruculose, with a thick, black and protruding scars at the base and macronematous conidiophores emerging from a poorly developed stroma. The protologue of *Ps. clematidis* only mentions 3–5-euseptate conidia at maturity in contrast to Ellis (1961), who reports up to 27-septate conidia in *E. ampullaceum*. However, some of the illustrated conidia (Fig. 37j p. 69) are distinctly rostrate and show up to 12 septa in agreement with most descriptions of *E. ampullaceum*. Rifai (2008), on the other hand, typified the genus *Neopodoconis* based on *E. ampullaceum*. Therefore, the material representing *Ps. clematidis* serves to place this genus in *Neomassarinaceae* (*Pleosporales*). Its acceptance is further supported by the distant placement of the generic type of *Exosporium*, currently *Helminthosporium tiliae* (syn. *Exosporium tiliae*), in *Massarinaceae* (Voglmayr & Jaklitsch, 2017). Accordingly, *Pseudohelminthosporium* is considered unnecessary and the following synonymy is proposed:

***Neopodoconis ampullacea*** (Petch) Rifai, *Reinwardtia* **12**(4): 278 (2008).

*Basionym*: *Helminthosporium ampullaceum* Petch, *Ann. R. bot. Gdns Peradeniya* 7(4): 319 (1922).

*Synonyms*: *Exosporium ampullaceum* (Petch) M.B. Ellis, *Mycol. Pap*. **82**: 32 (1961).

*Pseudohelminthosporium clematidis* Phukhams. & K.D. Hyde, *Fungal Diversity* **102**: 59 (2020).

### *Aquidictyomyces* is a synonym of *Biflagellospora*

Another recently introduced and putative novel genus, *Aquidictyomyces* (Dong et al. 2021), was based on an already described fungus. The generic type, *A. appendiculatus*, is clearly conspecific with the appendiculate species *Biflagellospora papillata* (Sivichai and Hywel-Jones 1999). Their conidia are identical in their distinct morphological appearance. They are composed of 8–9 cells, the main conidial body usually consisting of four cells with the two upper and larger ones having 4–8 distal ‘protuberances’ or ‘horns’ and bearing lateral arms which end in filiform, hyaline appendages up to 45 mm long. They also share a freshwater habitat and both fungi were collected from submerged wood in Thailand. The only difference between them is the polyblastic, sympodial conidiogenesis of *B. papillata* in contrast to the monoblastic, determinate conidiogenous cells observed in *A. appendiculatus*. However, the latter was based on the examination of a single specimen whereas *B. papillata* was described from two collections, which may better reflect the morphological and developmental variability of the fungus. Curiously, Fig. 30 g of the protologue of *A. appendiculatus* shows a different conidium consisting of two columns of cells with the distal ones ending in filiform appendages but lacking lateral arms or protuberances. This conidium corresponds to *B. japonica*, the generic type, originally described from Japan (Matsushima 1975) but also collected on submerged wood in different streams of Thailand (Sivichai and Hywel-Jones 1999). This contamination makes the phylogenetic placement of *A. appendiculatus* questionable and even despite the illustration of a germinating conidium (Fig. 30 h), it is unclear which fungus it belongs to. Therefore, further specimens and molecular data are necessary to confirm the position of *B. papillata* within the family *Annulatascaceae* (*Sordariomycetes*). Based on the available evidence, *Aquidictyomyces* is also considered redundant and the following synonymy is proposed:

***Biflagellospora papillata*** Sivichai & Hywel-Jones, *Mycol. Res.* **103**: 909 (1999).

*Synonym: Aquidictyomyces appendiculatus* W. Dong et al., *Mycosphere* **12**(1): 68 (2021).

### *Fusiconidium* is a synonym of *Camposporium*

Numerous genera of asexual *Ascomycota* are underrepresented by molecular data and “genotype first” identification inevitably leads to overlooking widely distributed and commonly occurring ones for which no sequences are available in public sequence databases. This is the case of *Endophragmiella* and the pleosporaceous genus *Fusiconidium*, recently described with two species (Li et al. 2017). Its diagnostic features such as well differentiated conidiophores proliferating percurrently and euseptate, fusiform conidia that secede rhexolytically match those of *Endophragmiella* whereas *Fusiconidium mackenziei*, the generic type, is clearly conspecific with *E. valdiviana*. This cosmopolitan species was originally described over a century ago as *Helminthosporium valdivianum* (Spegazzini 1910). Subsequently, it has been recombined into different genera and illustrated several times in major taxonomic contributions such as Ellis (1976), Hughes (1979) and Wu and Zhuang (2005). Morphologically, both fungi share the presence of fusiform to ellipsoidal, 7-septate conidia, broadly truncate at base and bearing a distinct basal frill as a result of their identical rhexolytic conidiogenesis. Conidiogenous cells proliferate percurrently 1–4 times whereas conidiophore and conidial dimensions overlap well although *F. mackenziei* has shorter conidia, 38–40 μm long. However, they are within the variability range of *E. valdiviana*, which has conidia that are reported as 28–58 μm long in Ellis (1963, 31–)34–42 μm in Hughes (1979). Moreover, the conidia of *F. mackenziei* show slight constrictions at the septa and this feature is also in agreement with the protologue of *E. valdiviana* (Spegazzini 1910). The only visible difference between them is that *F. mackenziei* apparently has echinulate conidia according to the protologue, although the original illustration (Fig. 1 p. 210) shows they are smooth.

Among species of *Endophragmiella*, only *E. taxi* and *E. dimorphospora* are represented in GenBank and their phylogenetic position is within *Helminthosporiaceae* (*Sordariales*) (Hernandez-Restrepo et al., 2017). *Fusiconidium mackenziei*, on the other hand, was placed within the distant *Melanommataceae* (*Pleosporales*). Therefore, *F. mackenziei* cannot be synonymized with *E. valdiviana* but a new combination should be provided. Hyde et al. (2020a) and Calabon et al. (2021) revealed that *Fusiconidium* grouped together with *Camposporium* but they considered these two genera distinct based on differences in conidiogenesis, conidial shape and the presence of apical appendages. From our point of view, however, there are numerous morphological similarities between them that justify their synonymy. Denticulate conidiogenous cells in *Camposporium* species are mono- or polyblastic, for example in *C. pellucidum* or *C. antennatum*. The narrow, cylindrical denticles function as separating cells involved in the rhexolytic secession of conidia. They are analogous to the slender, cylindrical and monoblastic conidiogenous cells associated with the rhexolytic detachment process seen in *F. aquaticum*, the second species of the genus (Li et al., 2017). Moreover, the majority of *Camposporium* species has cylindrical conidia with one or more appendages. In contrast, some others have fusiform conidia, e.g. *C. fusisporum* (Whitton et al., 2002) while others lack appendages (e.g. *C. ontariense, C. indicum*), being reminiscent of those of *Fusiconidium* species. The only distinctive feature between these genera is the proliferation of the conidiogenous cells, enteroblastic percurrent in *Fusiconidium* vs sympodial in *Camposporium*. However, *F. indicum* (Pratibha, et al., 2017) has polyblastic conidiogenous cells with sympodial proliferations and slender, denticle-like separating cells suggesting that this character is not significant in separating them. Nevertheless, accepting the percurrently proliferating *E. valdiviana* in *Fusiconidium* would make *Camposporium* paraphyletic according to the most recent phylogenies of Hyde et al., (2020a) and Calabon et al. (2021). Therefore, we refrain from doing so and provide a new combination in *Camposporium*.

***Camposporium valdivianum*** (Speg.) G. Delgado & Koukol, **comb. nov.**

MycoBank MB838825.

*Basionym*: *Helminthosporium valdivianum* Speg., *Revta Fac. Agron. Vet. Univ. nac. La Plata*, *Ser. 2* 6(1): 192 (1910).

*Synonyms: Endophragmiella valdiviana* (Speg.) S. Hughes, *N.Z. J. Bot.* **17**: 157 (1979).

*Fusiconidium mackenziei* Jun F. Li et al., *Phytotaxa* **308**(2): 211 (2017).

*Description:* Spegazzini (1910).

The other species described by Li et al. (2017) and Pratibha et al. (2017), *F. aquaticum* and *F. indicum*, respectively, are with no doubt also members of *Camposporium* based on the morphological and phylogenetic evidence explained above. Since new combinations in both cases would result in illegitimate later homonyms for *C. aquaticum* and *C. indicum*, replacement names are proposed instead. A fourth species, *F. lycopodiellae*, was already transferred to *Camposporium* by Hyde et al. (2020a).

***Camposporium verruculosum*** Koukol & G. Delgado, **nom. Nov.**

MycoBank: MB 838826.

*Replaced name*: *Fusiconidium aquaticum* Z.L. Luo & K.D. Hyde, *Phytotaxa* **308**(2): 211 (2017).

*Etymology*: Named after the verruculose conidia, a unique characteristic in *Camposporium*.

***Camposporium atypicum*** Koukol & G. Delgado, **nom. Nov.**

MycoBank MB 838827.

*Replaced name*: *Fusiconidium indicum* J. Pratibha & Prabhug., *Phytotaxa* **326**(2): 113 (2017).

*Etymology*: Referring to the atypical morphology of the slightly curved conidia, unusual in *Camposporium.*

### *Lylea tetracoila* is conspecific with *Sporidesmium lignicola*

Another example of neglecting existing literature while introducing a novel ascomycetous fungus is the description of *Sporidesmium lignicola* (Luo et al. 2019). This is the first *Sporidesmium* species having linked asexual and sexual morphs and it was described from decaying wood submerged in a freshwater stream in China. The asexual morph of *S. lignicola*, however, shows a strong morphological similarity to *Lylea tetracoila* (Hughes 1952; Ellis 1976; Holubová-Jechová 1978). Species of the asexually typified genus *Lylea*, based on *L. catenulata*, are characterized by cylindrical or fusiform, distoseptate conidia in unbranched, acropetal chains that emerge from monoblastic, determinate conidiogenous cells (Morgan-Jones, 1975; Xia et al., 2014). Both asexual *S. lignicola* and *L. tetracoila* form tufts of conidiophores similar in size, mostly 3-distoseptate, fusiform to cylindrical conidia 4.5–6.5 μm wide and arranged in long, unbranched acropetal chains. The only difference between them is that the conidia of *S. lignicola* are slightly shorter and may reach 21–27 μm long according to the protologue. However, a comparison with the scale bar in the original illustration (Fig. 20) indicates that conidia are longer and may reach 50 μm in length in agreement with previous descriptions of *L. tetracoila*. This fungus has a long history of different generic placements since it was originally described within the now dubious genus *Fusoma* more than a century ago (Corda, 1838). A well-documented species, it has been recorded and illustrated many times based on specimens collected throughout continental Europe (Holubová-Jechová 1978; Mel'nik 2000). It usually occurs on dead rotten wood, logs or fallen branches of different species of deciduous trees often growing on the ascomata or around the ostioles of pyrenomycetous fungi, mainly on the stromata of diatrypaceous ascomycetes. Alignment of unpublished ITS sequence from a Czech specimen (Fig. 1E–G) with strains of *L. tetracoila* deposited in CBS show they are identical. Similarly, alignment of LSU sequence with those belonging to the holotype and a paratype of *S. lignicola* was almost identical with only 3 out of 480 bps different. Therefore, these two species should be considered conspecific. A new combination is proposed based on the available morphological and molecular evidence:

***Sporidesmium tetracoilum*** (Corda) G. Delgado & Koukol, **comb. nov.**

MycoBank MB 838828.

*Basionym*: *Fusoma tetracoilum* Corda, *Icon. fung.* **2**: 5 (1838).

*Synonyms*: *Lylea tetracoila* (Corda) Hol.-Jech. *Folia Geobot. Phytotax.* **13**: 437 (1978).

*Sporidesmium lignicola* Z.L. Luo et al., *Fungal Diversity* **99**: 507 (2019).

*Descriptions*: Corda (1838) and Holubová-Jechová (1978).

*Specimen examined*: **Czech Republic**: Northern Bohemia, Bohemian Switzerland National Park, Mlýny above Vysoká Lípa, forest under the hilltop, on *Fagus* or *Acer* branch on the ground, 3 May 2018, *K. Prášil 4/2018* (PRC 4681, ITS-LSU sequence GenBank OU413153).

### The genus *Tricornispora* and *Eriosporella bambusicola*

The last cases we evaluate here are those of *Eriosporella* and the monotypic genus *Tricornispora*. Their phylogenetic placements were supposedly unknown until Dai et al. (2014) described *E. bambusicola* and provided a phylogenetic placement for *Eriosporella* within *Capnodiales*. However, the phylogenetic position of *Eriosporella* was known long before as *E. calami*, the generic type, was identified as the asexual morph produced in culture by *Lachnum nipponicum*, a member of *Lachnaceae* (*Helotiales*) (Haines & Kaneko, 1984). The fungus that Dai et al. (2014) examined was undoubtedly conspecific with *Tricornispora bambusae*. *Eriosporella bambusicola* was isolated from a similar bambusicolous substrate and the conidiogenous cells are polyblastic which also matches the concept of *Tricornispora* rather than *Eriosporella* (van der Aa & van Oorschot, 1985). Moreover, the conidial dimensions partially overlap. Conidial basal cells, for example, are 4.5–8.5 × 2.5–5 μm vs. 7–12 × 5 μm for *E. bambusicola* and *T. bambusae*, respectively, or nearly identical in the case of their conidial arms, 42.5–60 × 3–5 μm vs. 55–64 × 4–6 μm (Bonar 1967; Dai et al. 2014). The phylogenetic placement among *Capnodiales* thus refers to *T. bambusae*. Van der Aa and van Oorschot (1985) also mentioned the similarity between the conidia of *T. bambusae* with those of *Triglyphium bambusae*, which have shorter arms (15–49 × 3.3–5 μm) but still largely overlap. Considering also the identical type of conidioma and host, this species should be also treated as another synonym as follows:

***Tricornispora bambusae*** Bonar, *Mycologia* **59**: 597 (1967).

*Synonyms*: *Eriosporella bambusicola* D.Q. Dai et al., *Cryptog. Mycol*. **35**: 45 (2014).

*Triglyphium bambusae* A.K. Roy, *Sydowia* **20**: 203 (1968) [“1966”].

## CONCLUSIONS

Considering the large number of already described asexual fungi in *Ascomycota* still lacking molecular data, careful examination of past and present taxonomic literature continues to play a critical role in describing new taxa. This is especially relevant for old or well documented taxa to avoid re-describing them as new. Gams (2016) wisely pointed out this problem as “the tedious search in the literature for possibly available names for their isolate.” Although a full search of every relevant literature source and examination of all available fungal vouchers is not feasible (Hawksworth 2020), due diligence and conscious work from taxonomists in this regard is still strongly recommended. Fortunately, proper use of the many tools available nowadays including nomenclatural repositories and online accessible literature or databases opens up the possibility to keep the already huge taxonomic burden at a minimum.

Moreover, taxonomic decisions should be made responsibly following basic rules and recommendations provided regularly in informative and easy to follow articles (e.g. Aime et al. 2021). We would like to stress the importance of a thorough morphological study of multiple independent specimens leading to the recognition of interspecific variability in both molecular and phenotypic characteristics to produce a sound species hypothesis (Hawksworth 2020). Temptation to describe a taxonomic novelty based on a single gene or a single phenotypic difference should be overcome and robust support using multiple data should always be obtained. As in the case of a thorough morphological study, we also recommend using several molecular markers to provide stronger evidence for phylogenetic placement. Even those that are not ideal for phylogenetic analyses (e.g. due to excessive variability) should be amplified and published to allow a wider search in public databases and thus increase the probability of a match with an already published sequence. These rather simple steps will ensure the reliability of molecular data and obtained cultures and will provide an additional layer of quality control during fungal biodiversity studies.

## Data Availability

The only sequence obtained in this study was deposited in GenBank under Acc Nr. OU413153.
